# The Effect of Instrumental Timbre on Interval Discrimination

**DOI:** 10.1371/journal.pone.0075410

**Published:** 2013-09-16

**Authors:** Jean Mary Zarate, Caroline R. Ritson, David Poeppel

**Affiliations:** Department of Psychology, New York University, New York, New York, United States of America; University of California, Merced, United States of America

## Abstract

We tested non-musicians and musicians in an auditory psychophysical experiment to assess the effects of timbre manipulation on pitch-interval discrimination. Both groups were asked to indicate the larger of two presented intervals, comprised of four sequentially presented pitches; the second or fourth stimulus within a trial was either a sinusoidal (or “pure”), flute, piano, or synthetic voice tone, while the remaining three stimuli were all pure tones. The interval-discrimination tasks were administered parametrically to assess performance across varying pitch distances between intervals (“interval-differences”). Irrespective of timbre, musicians displayed a steady improvement across interval-differences, while non-musicians only demonstrated enhanced interval discrimination at an interval-difference of 100 cents (one semitone in Western music). Surprisingly, the best discrimination performance across both groups was observed with pure-tone intervals, followed by intervals containing a piano tone. More specifically, we observed that: 1) timbre changes within a trial affect interval discrimination; and 2) the broad spectral characteristics of an instrumental timbre may influence perceived pitch or interval magnitude and make interval discrimination more difficult.

## Introduction

The ability to perceive changing pitch in sounds is crucial for both speech and music. The contour of pitch changes in speech can determine the linguistic-communicative intent of a sentence (e.g., interrogative versus declarative versus imperative) and its affective content (happy, angry, sad, etc.), or—at the word level—distinguish lexical-semantic meanings in tonal languages [1]. The pitch contour in music outlines the melody. In more detail, the melodic contour can be subdivided into particular frequency ratios or intervals that have specific labels (and functions) in Western music composition, such as the minor third, perfect fifth, or the major seventh.

Since pitch intervals serve such a fundamental role in music, numerous studies have investigated the ability to discriminate pitch intervals in Western musical contexts (for a comprehensive review, see [2]). These experiments included tasks such as interval categorization or discrimination of interval magnitudes at or around musically relevant intervals [3], [4], [5], [6], [7], correcting mistuned intervals [8], [9], and assessment of performance intonation [10], [11]. It is likely that the explicitly musical contexts of these experiments—in which the discrimination tasks were based on musically relevant intervals—may have given musicians a significant advantage over non-musicians. In a recent experiment [12], we reduced the musical context by choosing frequencies that were not easily assigned to note names and interval magnitudes that are not often used in Western music (e.g., 25, 50, 75 cents), except one interval at 100 cents (a semitone). People with extensive musical expertise exhibited interval-discrimination thresholds of 100 cents, and non-musicians displayed larger thresholds [12], which: 1) corroborates McDermott et al.'s findings obtained with an adaptive procedure [13], and 2) suggests that an explicitly musical context in these studies may not influence the basic interval-discrimination thresholds in these groups. These thresholds may be established via repeated exposure to similar intervals in Western music and languages [14].

**Figure 1:**
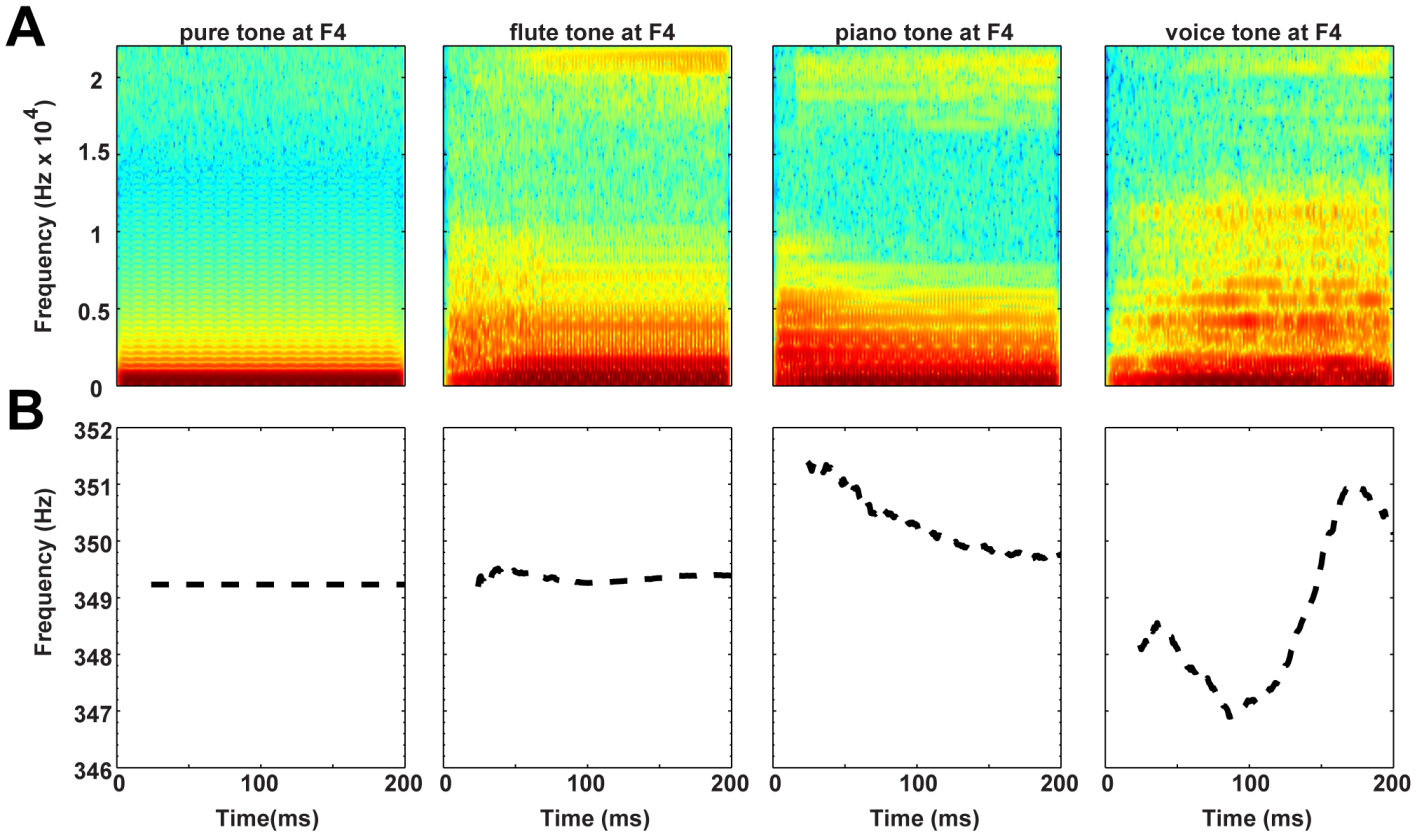
Spectrograms (A) and fundamental frequency traces (B) of the four tones at F4 (target base frequency of 349.23 Hz). The variations in spectral energy influence the estimated fundamental frequency for each of the instrumental tones.

While musically relevant frequencies or interval magnitudes may not affect interval-discrimination thresholds, changes in a tone's frequency spectrum—which creates a particular timbre—may influence pitch and/or interval perception (see Figure 1) [15], [16]; however, earlier experiments have yielded conflicting accounts of these effects in musicians and non-musicians. When music students were asked to tune musical intervals containing pure or synthetic complex tones, Rakowski determined that regardless of tone timbre, melodic intervals of a minor third or less are judged as even smaller in size than their actual pitch magnitude, and conversely larger intervals are perceived as bigger that their magnitude [17]. In contrast, Russo and Thompson found that timbre affected the perceived size of a melodic interval for both musicians and non-musicians, depending on whether synthetic timbre changed from a dull to a brighter sound (or the reverse manipulation) between the two tones [18]. Spiegel and Watson [19] and Micheyl et al. [20] reported that both musicians and non-musicians had better two-tone discrimination thresholds with synthetic complex tones than with pure tones; they argued that the enhanced frequency discrimination observed with complex tones, which are closer to real instrumental timbres that musicians hear during training, may be generalized to artificial pure tones. Demany and Semal suggested that this generalization is only partial, since they found that pitch discrimination abilities may be specific for the timbres used during training [21]. Finally, McDermott and his colleagues found that musicians and non-musicians had similar pitch- and interval-discrimination thresholds for both synthetic complex and pure-tone stimuli [13].

Given these disparate accounts of timbral effects on interval discrimination, we designed this study to assess the phenomenon employing different levels of controls. Most auditory discrimination studies have typically used pure or synthetic complex tones; here, we employed more naturalistic, instrument sound samples to assess the effects of timbre. Non-musicians and musicians were asked to indicate the larger of two presented intervals. We manipulated the timbre in one of the four presented stimuli (either pure, flute, piano, or synthetic voice) per trial; the other three stimuli were pure tones, as in a previous experiment [12]. We altered only one out of four tones to determine whether the introduction of a different timbre would alter the perceived pitch of a tone and, therefore, the perceived interval size. Additionally, we only changed one note per trial to prevent any confounding variables, such as interactions between two different non-sinusoidal timbres (one in each interval) and perceived pitch and/or interval size. Unlike our previous experiment, we sought to make this task more musically relevant by employing a base frequency (349.23 Hz or F4) and a large set of intervals that could be assigned to Western conventions: 100, 200, 300, 400, 500, 600 cents (i.e., the minor and major seconds, minor and major thirds, fourth, and the tritone). Based on earlier research [12], [13], [19], [20], [22], we predicted that the musicians would perform better on this discrimination task, due to their training-enhanced auditory skills. We also hypothesized—based on Spiegel and Watson's and Micheyl et al.'s reports of improved discrimination with complex tones—that instrumental timbres may improve all subjects' performances, since instrumental sounds are more naturalistic than pure tones that are only encountered in a laboratory setting. Finally, based on Demany and Semal's (2002) suggestion that enhanced auditory skills may be linked with exposure to specific timbres during musical training, musicians may exhibit greater improvement in interval discrimination during instrumental-timbre trials than non-musicians.

To summarize, we designed the present experiment to determine whether employing more naturalistic, instrumental timbres would improve interval discrimination in non-musicians and musicians, relative to only pure-tone stimuli. As detailed below, musicians discriminated between intervals better than non-musicians across all timbres, and interval discrimination was best with pure tones.

## Methods

### Ethics Statement

All testing was performed with the subjects' informed written consent and in accordance with procedures approved by the NYU University Committee on Activities Involving Human Subjects.

### Subjects

A total of 29 subjects were recruited from the New York University (NYU) community and surrounding areas. All subjects (mean age  = 24.8 years, SD = 6.56 years) were right-handed and had normal hearing. All subjects were categorized as non-musicians or musicians according to self-report of musical experience, as assessed by an in-house survey. Fourteen non-musicians (7 female) had minimal musical experience (mean  = 0.78 years, SD = 0.66 years) and did not play music regularly at the time of study. Fifteen musicians (7 female) had an average of 11.7 years of musical experience (SD = 5.83 years) and were practicing or performing music at the time of study. None of the subjects reported having absolute pitch.

### Stimuli

We used MATLAB (Mathworks, Natick, MA, USA) to create sinusoidal tones at a base frequency of 349.23 Hz, which corresponds to an F4 in Western music. The instrument sounds (piano, flute, voice) were MIDI-generated (Musical Instrument Digital Interface) from a Yamaha YPT-220 keyboard (Yamaha Corporation of America, Buena Park, CA, USA) at this same base frequency. All instrument sounds included the attack (or onset) of the sound and had no vibrato. Additional sinusoidal and instrumental tones were generated at specific pitch distances—50 to 600 cents at 25-cent increments—from this base frequency. All tones (200-ms duration, 16-bit depth, 44100-Hz sampling frequency) were gated with 7-ms cosine ramps in MATLAB and then normalized to 0.8 dB in Audacity (open-source freeware, http://audacity.sourceforge.net/). Figure 1 displays the spectrograms and the fundamental frequency (F0) estimated with YIN [23] for each timbre at F4. Compared to the pure tone, all instrumental timbres have more energy across a broader span of frequencies (including harmonics of the base frequency). Additionally, the flute and synthetic voice tones have more diffuse onsets, relative to the sharper onsets of the piano and pure tones. Finally, the frequency range of spectral energy increases from the piano to the flute tones, with the synthetic voice timbre displaying the broadest frequency distribution of energy compared to all other tones. The various changes in spectral energy apparently result in slight fluctuations of estimated F0 in the piano and synthetic voice timbres (Figure 1B).

In MATLAB, tones were paired with a 50-ms gap of silence in between to create interval sizes ranging from 50 to 600 cents (respectively, a quarter-tone to a tritone in Western music), and intervals were combined (ISI = 0.8, 0.9, or 1 s) to create individual test trials. We parametrically varied the magnitude differences between intervals within a trial from 0 to 100 cents (a semitone in Western music) in 25-cent increments. We chose 100 cents as the maximum interval-difference due to both its musical relevance and the observation that musicians' performances approach a ceiling of maximum accuracy at around this magnitude [12]. Zero-cent differences between intervals in a trial were included to observe whether subjects—when forced to guess— had a response bias based on timbre type. For all trials, the first tone of each interval was a pure tone at the base frequency of 349.23 Hz. In trials with an instrumental timbre, the instrument sound could occur either as the second or the fourth tone.

### Experimental procedure

In a sound-attenuated booth, subjects sat in front of a lab computer and wore headphones (Sennheiser HD 380 Professional, Sennheiser Electronic Corporation, Wedemark, Germany), through which all auditory stimuli were delivered via MATLAB at a comfortable level (∼77.5 dB SPL). A 10-trial demonstration was presented at the beginning of the session to familiarize subjects with the different sounds presented during the experiment. Prior to interval discrimination, pitch-discrimination thresholds were determined with pure tones at 349.23 Hz in a “2 down – 1 up” staircase procedure [24], implemented as part of the MLP toolbox for auditory psychophysical testing [25]. After discrimination-threshold testing, subjects were presented with intervals in a two-alternative forced-choice design, indicating by button press which pair contained the larger interval. Subjects received visual feedback (“correct” or “incorrect”) on the computer screen after making each decision. There were at least 15 trials of each interval type (pure, flute, piano, and voice) for each interval-difference, all presented in a pseudo-randomized order. In total, there were 5 blocks with 100 trials each, and subjects were allowed to take a short break between blocks.

### Analyses

Subjects' performances were measured as percent-correct scores for each of the interval-differences (25–100 cents). We also calculated *d*-prime and *β*normalized values to measure detector sensitivity and response bias, respectively [26], [27], [28]. The hit and false alarm (FA) rates, *d*-prime (*d′*), and *β*normalized values were calculated as follows:

Hit  = H(# times 1^st^ pair was chosen/# trials with larger 1^st^ pair)  =  score for 1^st^-pair trials.
FA  = 1 – H(# times 2^nd^ pair was chosen/# trials with larger 2^nd^ pair)  = 1 – score for 2^nd^-pair trials.
*d′*  =  Zscore(Hit) – Zscore(FA)
*β* = −0.5*(Zscore(Hit) + Zscore(FA))
*β*_normalized_  =  *β*/*d′*; 0 =  no bias; negative values  =  bias towards selecting 1^st^ pair; positive values  =  bias towards selecting 2^nd^ pair.

For equal-interval trials (0-cent difference between intervals), response bias was calculated as a proportion of the total number of trials in which subjects selected the interval with an instrumental timbre, instead of the pure-tone interval; higher proportions reflect a stronger bias towards selecting the instrumental-timbre intervals.

Pitch-discrimination thresholds (in cents), percent-correct scores, *d′* values, *β*normalized values, mean reaction time, and standard deviation of reaction times were analyzed using an independent-samples *t*-test or repeated-measures analyses of variance (ANOVA). Tukey's Honestly Significant Difference test and planned comparisons were used for post-hoc analyses of significant main effects and interactions, respectively.

## Results

### Effects of musical expertise on pitch-discrimination thresholds

An independent samples *t*-test performed on the musicians' and non-musicians' pitch-discrimination thresholds measured at F4 (349.23 Hz) determined that musicians' thresholds (mean ± SEM  = 14.6±2.7 cents) were significantly lower than non-musicians' thresholds (44.1±9.7 cents) as expected [*t*(27)  = 1.22, *p*<0.05].

### Effects of musical expertise and timbre on interval-discrimination accuracy

**Figure 2:**
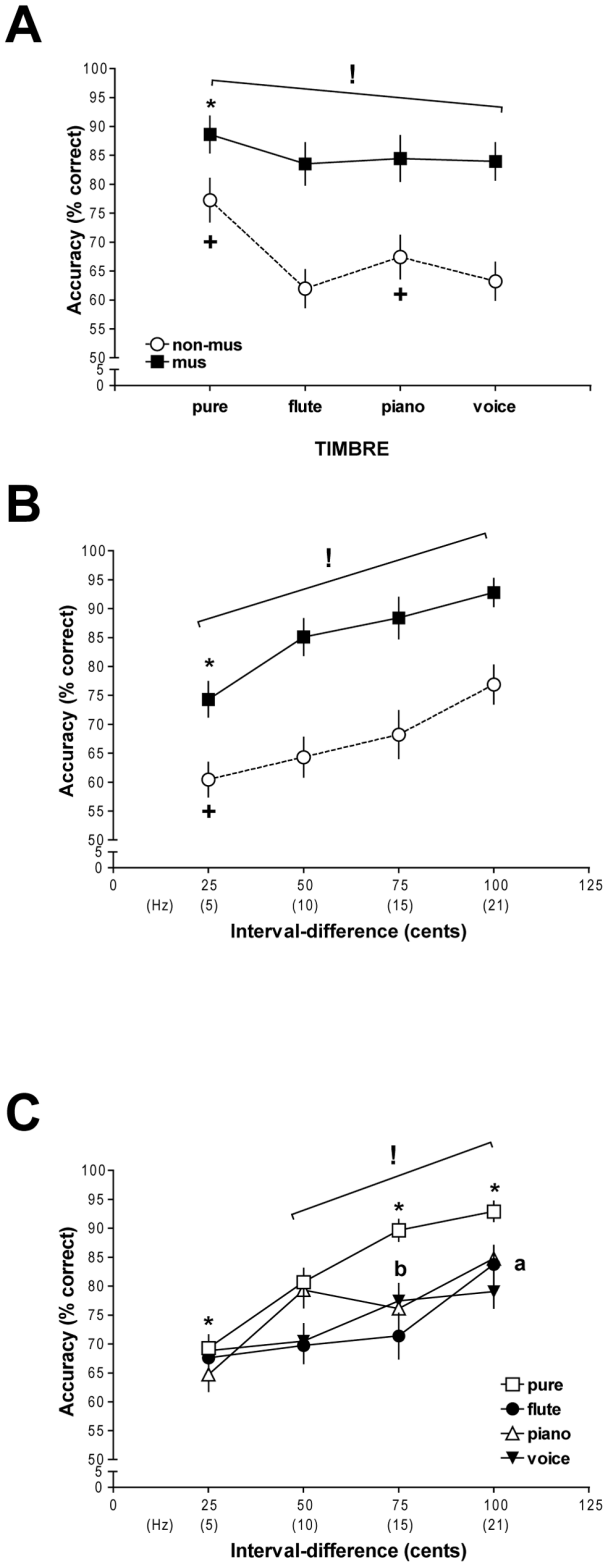
Interval-discrimination accuracy. (A) Mean ± SEM percent-correct scores of musicians and non-musicians for interval discrimination, across the four timbre types and collapsed across all interval-differences. Musicians performed more accurately than non-musicians across all timbres (indicated by**!**, *p*<0.001), but were marginally most accurate with pure-tone intervals (denoted by *, *p*<0.06). Non-musicians showed more accurate interval discrimination with pure and piano tones than with the other two timbres (marked by **+**, *p*<0.05). (B) Mean ± SEM percent-correct scores of musicians and non-musicians for each interval-difference collapsed across timbre types. Musicians were more accurate than non-musicians overall (marked by**!**, *p*<0.001). Both musicians (denoted by *, *p*<0.001) and non-musicians (indicated by **+**, *p*<0.05) displayed the least accuracy at the 25-cent interval-difference. (C) Mean ± SEM percent-correct scores for each timbre type at each interval-difference, averaged across both groups. All subjects discriminated pure-tone intervals more accurately than flute- and voice-tone intervals at all interval-differences except 25 cents (shown by**!**, *p*<0.001), and better than piano-tone intervals at all interval-differences except 50 cents (marked with *, *p*<0.06). Flute-tone discrimination only improved at 100 cents (shown by **a**, *p*<0.001), while voice-tone performance significantly improved at interval-differences of 75 cents and higher (indicated by **b**, *p*<0.01).

Figure 2 depicts the significant results of a three-way repeated-measures ANOVA performed on percent-correct scores, with group as the between-subject factor and timbre and interval-difference as the repeated within-subject factors. The analysis revealed significant main effects of group [*F*(1,27)  = 33.64, *p*<0.001], timbre [*F*(3,81)  = 22.87, *p*<0.001], and interval-difference [*F*(3,81)  = 88.47, *p*<0.001], and significant two-way interactions between group and timbre [*F*(3,81)  = 5.75, *p*<0.01], group and interval-difference [*F*(3,81)  = 4.65, *p*<0.01], and timbre and interval-difference [*F*(9,243)  = 8.44, *p*<0.001]. No other interactions were significant.

Planned comparisons performed on the group-by-timbre interaction determined that musicians discriminated intervals of all timbres more accurately than non-musicians (Figure 2A; *p*s<0.001), which reiterated the significant group main effect, but musicians discriminated intervals with all pure tones marginally better than with other timbres (*p*s<0.06). Non-musicians on the whole performed more accurately with pure and piano tones, which may be due to their sharper onsets and/or relatively compact distribution of sound energy (see Figure 1), compared to flute and synthetic voice timbres (*p*s<0.05). Planned comparisons on the two-way interaction between group and interval-difference showed that musicians discriminated between intervals more accurately at all interval-differences than non-musicians (Figure 2B; *p*s<0.001). Both groups showed a significant, steady improvement in accuracy as interval-differences increased, and had the worst discrimination accuracy at the 25-cent interval-difference compared to all other magnitudes (*p*s<0.001 for musicians; *p*s<0.05 for non-musicians).

Planned comparisons on the timbre-by-interval-difference interaction determined that discrimination with pure-tone intervals significantly improved at each larger interval-difference (Figure 2C; *p*s<0.05). Pure-tone discrimination was also better than with flute- and voice-tone intervals at all interval-differences except 25 cents (*p*s<0.001), and marginally more accurate than with piano-tone intervals at all interval-differences other than 50 cents (*p*s<0.06). This suggests that in general, interval discrimination with pure tones was better than with any other timbre. Performance with flute tones only significantly improved at the 100-cent interval-difference (*p*s<0.001). Discrimination accuracy with piano-tone intervals improved as interval-differences increased (*p*s<0.05), but with no significant change in accuracy between interval-differences of 50 and 75 cents. Voice-tone interval discrimination improved significantly beginning at the 75-cent interval-difference (*p*s<0.01); there were no significant changes in accuracy between 25- and 50-cent interval-differences and between 75- and 100-cent interval-differences. Notably, voice-tone interval discrimination was worse at 100 cents than with any other timbre (*p*s<0.01).

### Effects of musical expertise and timbre on interval-discrimination sensitivity

**Figure 3:**
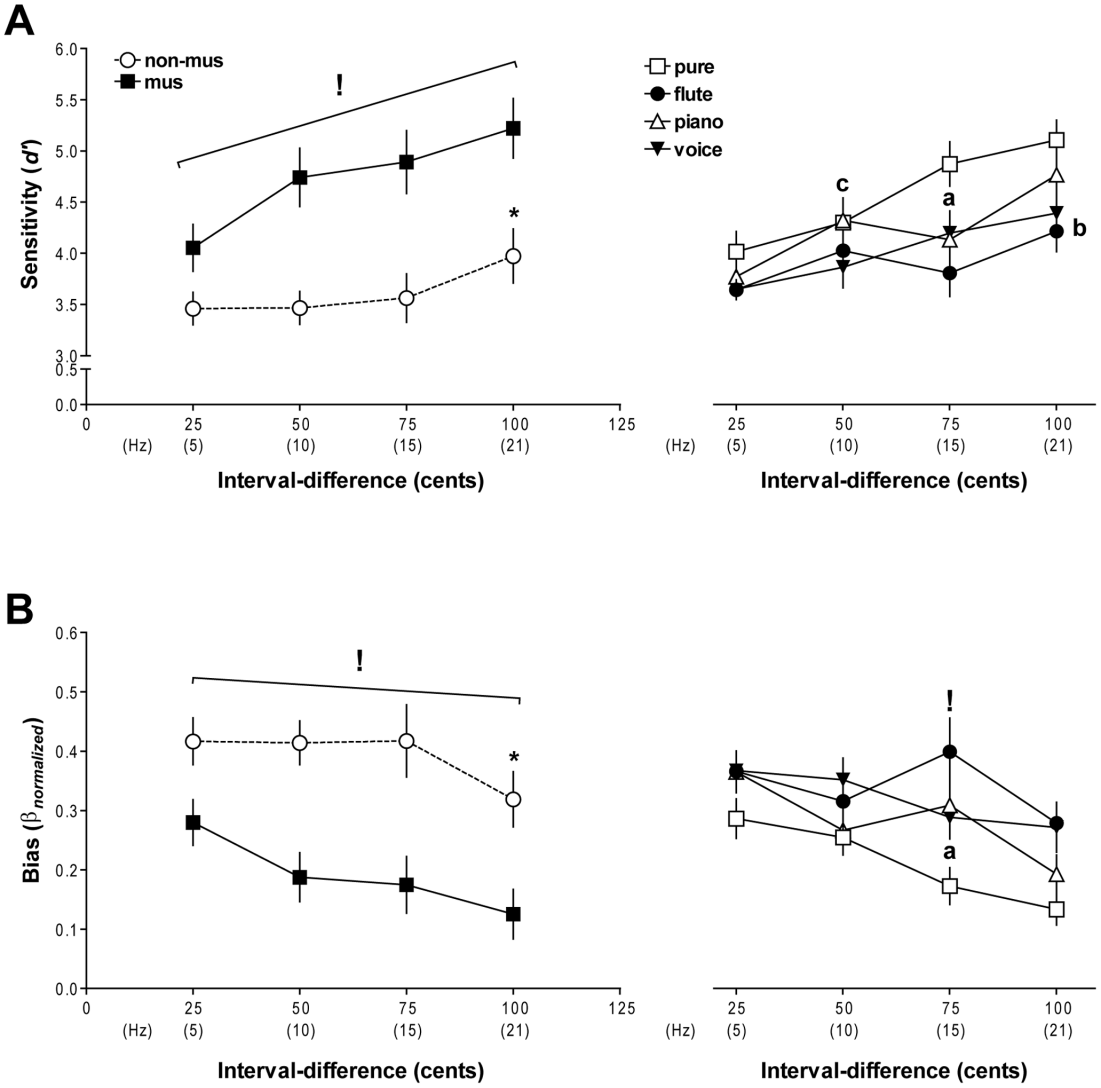
Interval-discrimination sensitivity and response bias. (A) Left: Mean ± SEM *d′* values of musicians and non-musicians across all timbres at each interval-difference. Musicians exhibited more discrimination sensitivity across all interval-differences, compared to non-musicians (denoted by**!**, *p*<0.01). Across interval-differences, on-musicians only showed enhanced discrimination sensitivity at a difference of 100 cents (marked by *, *p*<0.01). Right: Mean ± SEM *d′* values for each timbre at each interval-difference, averaged across both groups. Listeners exhibited enhanced discrimination sensitivity at interval-differences of 75 cents with pure and voice tones (indicated with **a**, *p*s<0.05), 100 cents with flute tones (marked with **b**, *p*<0.001), and 50 cents with a piano timbre (denoted by **c**, *p*<0.06). (B) Left: Mean ± SEM *β_normalized_* values (measure of response bias) for musicians and non-musicians at each interval-difference, collapsed across all timbre types. In general, non-musicians showed greater bias towards selecting the second interval as the larger interval than non-musicians (shown with**!**, *p*<0.01), and this bias only significantly decreased within the group at the 100-cent interval-difference (indicated by *, p<0.001). Right: Mean ± SEM *β_normalized_* values for each timbre at all interval-differences, averaged across both groups. Response bias decreased significantly with pure and voice-tones at 75 cents (marked by **a**, *p*s<0.05). Interestingly, response bias was highest at a 75-cent interval-difference with flute tones, compared to all other timbres (shown by**!**, *p*<0.05).

Figure 3A shows the results of a three-way repeated-measures ANOVA (group by timbre by interval-difference) performed on *d′* prime values to assess the influence of musical expertise on interval-discrimination sensitivity. The analysis resulted in significant main effects of group [*F*(1,27)  = 23.28, p<0.001], timbre [*F*(3,81)  = 12.23, *p*<0.001], and interval-difference [*F*(3,81)  = 28.04, *p*<0.001], and significant two-way interactions between group and interval-difference [*F*(3,81)  = 7.02, *p*<0.001] and timbre and interval-difference [*F*(9,243)  = 2.47, *p*<0.05]. Planned comparisons on the group-by-interval-difference interaction determined that musicians displayed more discrimination sensitivity than non-musicians across all interval-differences (Figure 3A, left; *p*s<0.01), as reflected by the main group effect. Among musicians, sensitivity improved as a function of increasing interval-difference (*p*s<0.01) irrespective of timbre, with the exception of no significant change in sensitivity between interval-differences of 50 and 75 cents. Within the non-musician group, discrimination sensitivity was only significantly enhanced at an interval-difference of 100 cents (*p*s<0.01); there were no other changes in sensitivity seen among smaller interval-differences (*p*s>0.5).

Planned comparisons on the interaction between timbre and interval-difference revealed that *d′* values increased significantly across both groups for both pure- and voice-tone interval discrimination at interval-differences of 75 cents and larger (Figure 3A, right; *p*s<0.05). The *d′* values were higher for pure-tone interval discrimination than for flute-tone discrimination at all interval-differences (*p*s<0.05) except 50 cents, piano-tone discrimination at only 75 cents (*p*<0.001), and discrimination with voice tones overall (*p*s<0.05). Among the instrumental timbres, sensitivity for piano tones was marginally higher at 50–100 cents than for flute tones (*p*s<0.08) and at 50- and 100-cent interval-differences than for voice tones (*p*s<0.07). Sensitivity to flute-tone intervals increased significantly between 25 and 50 cents (*p*<0.01) and at the 100-cent interval-difference (*p*s<0.001). The *d′* values for piano-tone discrimination marginally increased at interval-differences of 50 cents and higher (*p*s<0.06), with no significant changes in sensitivity between 50- and 75-cent interval-differences.

Overall, interval-discrimination performance was best with pure-tone intervals, except at the smallest interval-differences of 25 cents; changes in timbral qualities did not enhance interval discrimination at this very small magnitude. Discrimination sensitivity was poorest with flute tones, whereas the poorest accuracy was observed with voice-tone intervals—accuracy with voice tones was still significantly lower than all other timbres at the 100-cent interval-difference. The discrepancy between accuracy and discrimination sensitivity may be explained by response bias, as discussed below.

### Effects of musical expertise and timbre on response bias during interval discrimination

Figure 3B shows results from a three-way repeated measures ANOVA (group by timbre by interval-difference) on *β_normalized_* values to determine the effects of musical training and timbre on response bias. We found significant main effects of group [*F*(1,27)  = 21.70, *p*<0.001], timbre [*F*(3,81)  = 16.70, *p*<0.001], and interval-difference [*F*(3,81)  = 27.89, *p*<0.001], as well as significant group-by-interval-difference [*F*(3,81)  = 5.61, *p*<0.01] and timbre-by-interval-difference interactions [*F*(9, 243)  = 3.57, *p*<0.001].

Planned comparisons on the group-by-interval-difference interaction revealed that non-musicians showed greater bias than musicians towards choosing the second interval as the larger interval across all interval-differences (Figure 3B, left; *p*s<0.01), as also indicated by the significant group main effect. Musicians' response bias decreased as interval-differences grew (*p*s<0.05), except for no change in response bias between interval-differences of 50 and 75 cents. Among non-musicians, response bias only significantly decreased at the 100-cent interval-difference (*p*s<0.001); no other significant changes in bias were seen at smaller magnitudes (*p*s>0.8). This result is mirrored by non-musicians' significant increase in discrimination sensitivity at only a 100-cent interval-difference (see results for *d′* values above).

Planned comparisons performed on the timbre-by-interval-difference interaction determined that response bias significantly decreased with pure tones as interval-differences increased to 75 cents and larger (Figure 3B, right; *p*s<0.05). There was also less response bias with pure-tone intervals compared to flute and voice tones at all interval-differences (*p*s<0.05), and relative to piano tones at 25- and 75-cent interval-differences (*p*s<0.01). During interval discrimination with flute tones, response bias was higher at interval-differences of 25 and 75 cents than at 50 and 100 cents (*p*s<0.05); notably, bias unexpectedly increased between interval-differences of 50 and 75 cents (*p*<0.05). The bias observed at 75 cents with flute tones was the largest compared to all other timbres (*p*s<0.05), which may explain why sensitivity was worst with flute tones and only improved at a 100-cent interval-difference. Response bias with piano-tone intervals reduced as a function of increasing interval-difference (*p*s<0.05), with the exception of no significant bias change between interval-differences of 50 and 75 cents. During interval discrimination with voice tones, response bias decreased significantly at 75 cents and larger (*p*s<0.05); bias did not change between 25- and 50-cent and between 75- and 100-cent differences between intervals.

To assess whether timbre influenced response bias with equal-magnitude intervals as subjects were forced to guess the larger interval, we performed an additional three-way repeated measures ANOVA on response-bias scores from the 0-cent interval-difference trials; response bias was measured as the proportion of trials in which subjects selected the interval with an instrumental timbre instead of the pure-tone interval. The analysis resulted in a significant group effect [*F*(1,27)  = 16.10, *p*<0.001]—non-musicians exhibited a greater bias (mean ± SEM: 0.66±0.04) than musicians (0.53±0.03) towards selecting intervals with instrumental timbres when there was no difference in interval magnitude.

### Effects of musical expertise and timbre on reaction time during interval discrimination

**Figure 4:**
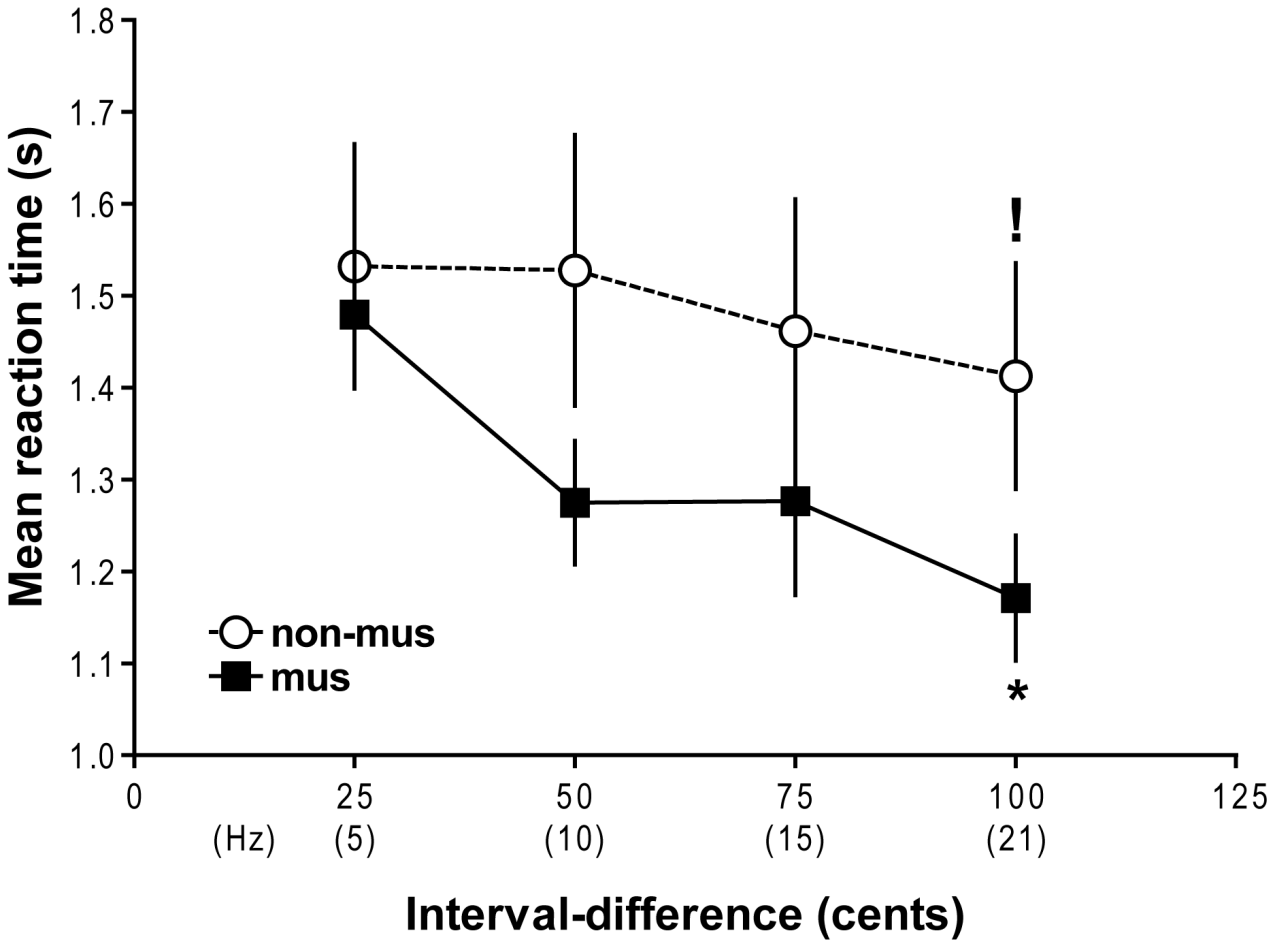
Mean ± SEM reaction times (in seconds) for musicians and non-musicians, averaged across all timbres. Musicians answered marginally faster than non-musicians at a 100-cent interval-difference (marked by *, *p*<0.09). Within the non-musician group, reaction times were faster at an interval-difference of 100 cents, compared to 25 and 50 cents (indicated by**!**, *p*<0.01).

Figure 4 displays results from analyses on mean reaction times obtained during interval discrimination. We found significant main effects of timbre [*F*(3,81)  = 4.68, *p*<0.01] and interval-difference [*F*(3,81)  = 23.77, *p*<0.001], and a significant group-by-interval-difference interaction [*F*(3,81)  = 6.40, *p*<0.001]. Post-hoc tests performed on the timbre main effect determined that all subjects responded marginally faster during interval discrimination with pure tones than any other timbre (Table 1; *p*s<0.06). Planned comparisons on the group-by-interval-difference interaction revealed that musicians were marginally faster than non-musicians in discriminating intervals that were 100 cents apart (Figure 4; *p*<0.09), but not at any other interval-difference. Within the musician group, reaction times reduced as interval-differences increased (*p*s<0.01), except for no significant change in reaction time between interval-differences of 50 and 75 cents. Non-musicians responded more quickly at interval-differences of 100 cents than at 25 and 50 cents (Figure 4; *p*s<0.01).

**Table 1:** Mean ± SEM reaction times (in seconds) during interval discrimination with each timbre type.

Timbre	Mean Reaction Time ± SEM (s)
pure	1.329±0.066*
flute	1.428±0.089
piano	1.400±0.088
voice	1.399±0.078

Both musicians and non-musicians discriminated between pure-tone intervals faster than intervals with other timbres (marked by *, *p*<0.06), regardless of interval-difference.

Analyses of the variability of reaction time (measured as a subject's standard deviation of reaction time) revealed a marginally significant main effect of timbre [*F*(3,81)  = 2.20, *p*<0.1]; post-hoc tests determined that response times were marginally more variable during flute-tone discrimination than during pure-tone discrimination (Table 2; *p*<0.08); no other significant differences were found. Based on accuracy and sensitivity scores, interval discrimination with flute tones may be more difficult, and this may be accompanied by more variable response times.

**Table 2:** Average variability ± SEM of reaction time (in seconds) for each timbre type.

Timbre	Mean Variability of Reaction time ± SEM (s)
pure	0.540±0.060
flute	0.720±0.146 *
piano	0.669±0.177
voice	0.607±0.073

Across all interval-differences, all subjects showed more variable response times during interval discrimination with flute tones than with pure tones (indicated by *, *p*<0.08).

Analyses of the reaction times during the equal-interval trials resulted in a significant timbre effect [*F*(3,81)  = 4.06, *p*<0.01]—all subjects responded more quickly during flute-tone trials than during pure- and voice-tone trials (Table 3; *p*s<0.05), but not compared to during piano-tone trials. Analyses of the variability of reaction time during equal-interval trials did not reveal any significant other main effects or interactions (*p*s>0.2).

**Table 3:** Mean ± SEM of reaction time (in seconds) during equal-interval trials.

Timbre	Mean Reaction Time ± SEM (s)
pure	1.625±0.089
flute	1.469±0.068 *
piano	1.578±0.091
voice	1.600±0.088

Regardless of interval-difference, flute-tone intervals elicited shorter reaction times from all subjects, compared to intervals with pure and voice tones (shown with *, *p*<0.05).

## Discussion

As expected, musicians discriminated between intervals more accurately than non-musicians across all interval-differences. In general, accuracy (as measured by percent-correct scores) improved in both groups as interval-differences increased. When examining discrimination sensitivity (represented by *d′* values), musicians displayed a significant increase in discrimination sensitivity across most interval-differences, while non-musicians exhibited an interval-discrimination sensitivity threshold of 100 cents. Originally, we hypothesized that instrumental sounds would enhance interval-discrimination performance in all subjects, relative to pure tones. However, although non-musicians displayed a stronger bias to select the interval with any instrumental sound among pairs of equal-magnitude intervals, both groups typically discriminated between intervals better and faster with pure tones than with any of the instrumental timbres; this evidence contradicts Micheyl and colleagues' report of enhanced auditory perception with complex timbres relative to pure tones [20]. Among the instrumental timbres, interval discrimination was best with piano tones (perhaps due to a sharper onset or attack), and intervals with flute tones (with diffuse tone onsets) elicited arguably the worst discrimination sensitivity.

Since both groups also took longer to respond during instrumental-timbre trials, it is possible that changing the timbre of only one out of four stimuli presented within a trial could have caused a distraction during discrimination; sequential tones with different timbres may be difficult to group together as intervals in our experiment [29]. Indeed, a previous study has suggested that the introduction of a new timbre can violate expectations that each successive sound will match the timbre of the previous one(s); in that study, this violation of expectation manifested as decreased discrimination accuracy [30]. Our observed interaction between timbre changes and decreased task performance is supported by Borchert et al.'s observations [31]: subjects had difficulty discriminating between two sequentially presented tones with different timbres, compared to two simultaneous tones with different timbres. Moreover, timbre seems to have a stronger interaction with pitch extraction/judgment and the evaluation of interval size when there is no tonal context (as in our interval-discrimination task), as opposed to tasks with a tonal reference point (or “key”) [30]. In fact, pitch changes seem to be best perceived (regardless of timbre) when F0 changes by at least 4%; the perception of smaller F0 changes is more influenced by timbre changes [16]. In our task, two interval sizes and interval-differences have F0 changes of less than 4% of the base frequency (i.e., 25 and 50 cents from 349.23 Hz). Thus, our experimental design perhaps allowed for a stronger influence of timbral manipulation on pitch perception and interval discrimination than expected, which may have rendered interval comparisons with instrumental timbres more difficult. This may also explain the non-musicians' bias towards selecting the interval with an instrumental timbre as the larger interval, even when the interval magnitudes were equal; timbral changes may have altered non-musicians' perception of the instrumental tones' spectral centroid and consequently the interval magnitude (see [18]). In contrast, musicians are reported to be less susceptible to timbre-based illusions of interval size, making them less likely to perceive the instrumental interval as larger [18].

Although we expected an improvement in interval discrimination based on both instrumental timbre and musical expertise, such an interaction was not observed in this study. However, when comparing the present results to those of our previous study with only pure-tone intervals [12], the use of instrumental timbre with intervals based on a musically relevant frequency appears to improve interval discrimination in each group, specifically among our parametrically varied interval-differences. In our earlier study, we reported pure-tone interval-discrimination thresholds of 100 cents in musicians, and 150 cents in non-musicians, which echoed McDermott et al.'s (2010) findings with both pure- and complex-tone intervals. Surprisingly, the musicians enrolled in this study did not display a threshold in interval discrimination (when averaging across all timbres), but rather a steady, significant increase in performance as the interval-differences grew larger. In addition, the non-musicians here demonstrated a smaller interval-discrimination threshold of 100 cents (across all timbres), rather than the threshold seen in our earlier experiment with only pure tones. In general, these qualitative performance changes across the two studies suggest that timbre specifically from musical instruments may improve or aid interval discrimination. Whether this instrumental-timbre effect can be disentangled from the effect of implementing a musically relevant base frequency (F4), rather than the base frequencies employed in our earlier study or in McDermott et al.'s (2010) study, must be explored further in later research.

However, these interpretations of timbral effects should be taken cautiously, since the best interval discrimination was still observed with pure-tone stimuli, which may have been due partly to higher presentation rates of pure-tone intervals throughout the experiment, compared to instrumental-tone intervals; a practice effect with this particular timbre may have enhanced pure-tone interval discrimination. Nevertheless, compared to previous conflicting accounts of the timbral effects on interval discrimination, we observed that: 1) changes from one timbre to another, especially within the same trial, significantly affect interval discrimination, and 2) the varied spectral energy of instrumental timbre can alter pitch perception and/or interval discrimination.
